# Symptom clusters among cancer survivors: what can machine learning techniques tell us?

**DOI:** 10.1186/s12874-021-01352-4

**Published:** 2021-08-16

**Authors:** Koen I. Neijenhuijs, Carel F. W. Peeters, Henk van Weert, Pim Cuijpers, Irma Verdonck-de Leeuw

**Affiliations:** 1 Department of Clinical, Vrije Universiteit Amsterdam, Neuro- and Developmental Psychology, Amsterdam Public Health Research Institute, Van der Boechorststraat 1, 1081 BT Amsterdam, The Netherlands; 2 Amsterdam UMC, Cancer Center Amsterdam, Amsterdam, The Netherlands; 3 Department of Epidemiology & Biostatistics, Amsterdam UMC, location VUmc, Boelelaan, 1117 Amsterdam, The Netherlands; 4 Mathematical & Statistical Methods Group (Biometris), Wageningen University & Research, Wageningen, The Netherlands; 5 Department of General Practice, Amsterdam UMC, location AMC, Amsterdam Public Health, Meibergdreef 9, Amsterdam, The Netherlands; 6 Department of Otolaryngology-Head and Neck Surgery, Amsterdam UMC, location VUmc, Boelelaan, 1117 Amsterdam, The Netherlands

**Keywords:** Cancer, Oncology, Symptom clusters, Machine learning

## Abstract

**Purpose:**

Knowledge regarding symptom clusters may inform targeted interventions. The current study investigated symptom clusters among cancer survivors, using machine learning techniques on a large data set.

**Methods:**

Data consisted of self-reports of cancer survivors who used a fully automated online application ‘Oncokompas’ that supports them in their self-management. This is done by 1) monitoring their symptoms through patient reported outcome measures (PROMs); and 2) providing a personalized overview of supportive care options tailored to their scores, aiming to reduce symptom burden and improve health-related quality of life. In the present study, data on 26 generic symptoms (physical and psychosocial) were used. Results of the PROM of each symptom are presented to the user as a no well-being risk, moderate well-being risk, or high well-being risk score. Data of 1032 cancer survivors were analysed using Hierarchical Density-Based Spatial Clustering of Applications with Noise (HDBSCAN) on high risk scores and moderate-to-high risk scores separately.

**Results:**

When analyzing the high risk scores, seven clusters were extracted: one main cluster which contained most frequently occurring physical and psychosocial symptoms, and six subclusters with different combinations of these symptoms. When analyzing moderate-to-high risk scores, three clusters were extracted: two main clusters were identified, which separated physical symptoms (and their consequences) and psycho-social symptoms, and one subcluster with only body weight issues.

**Conclusion:**

There appears to be an inherent difference on the co-occurrence of symptoms dependent on symptom severity. Among survivors with high risk scores, the data showed a clustering of more connections between physical and psycho-social symptoms in separate subclusters. Among survivors with moderate-to-high risk scores, we observed less connections in the clustering between physical and psycho-social symptoms.

## Introduction

Cancer survivors experience a myriad of symptoms rooted in physiology caused by the disease itself or caused by the treatment thereof [1]. Problems in the psychosocial domain are also prevalent [2–4]. Many of these symptoms and problems may co-occur and are likely interrelated. For example, sleep problems have been identified as both a risk factor and as a symptom of depression in both cancer [5] and non-cancer populations [6]. In cancer patients, subjective cognitive functioning has been associated with depression, anxiety, and fatigue [7]. Furthermore, problems with sexual health have been related to body image issues, depression, and anxiety [8]. Fatigue has been associated with pain, sleep issues, and depression; and nausea with vomiting [9].

Such interrelated symptoms are referred to as symptom clusters, and knowledge regarding such symptom clusters may inform targeted interventions [10]. Some studies have set out to empirically determine symptom clusters using various types of cluster analyses. In 2011, a systematic review identified 47 studies that statistically investigated symptom clusters in cancer patients [9]. A number of symptom clusters repeatedly showed up: (i) a fatigue-depression-pain cluster, (ii) a nausea-vomiting cluster, and (iii) a depression-anxiety-insomnia cluster. However, the authors note that these (and other) clusters seem heavily influenced by the population studied (tumor type, cancer stage, treatment modality and treatment intent), symptom assessment method, and statistical method used. Also, many of these studies were limited in scope in terms of sample size, number of symptoms investigated, or the type of analysis that was used.

Another systematic review was performed for studies up to 2016 which focused on cancer patients receiving primary or adjuvant chemotherapy [11]. Nineteen studies were included, and a few consistently appearing symptom clusters were identified: (i) a nausea-vomiting cluster, (ii) a psychological symptom cluster, and (iii) a “sickness behavior” (pain-fatigue-insomnia-lack of appetite) cluster. Noteworthy is that the individual symptoms in each of these clusters were not necessarily consistent between studies.

In 2015 an international expert panel regarding “Advancing Symptom Science Through Symptom Cluster Research” was formed [10]. This panel was subdivided into five groups: (i) defining characteristics of symptom clusters, (ii) identification of priority symptom clusters and underlying mechanisms, (iii) measurement of symptom clusters, (iv) targeted interventions for symptom clusters, and (v) new analytic strategies for symptom cluster research. In line with the aforementioned review [9], the first expert group concluded that there is little consistency in the number and types of symptom clusters identified in cancer patients/survivors. This expert group defined a number of directions for future research in defining symptom clusters. In particular, they stated a need for “the establishment of a common conceptual framework and approach for the evaluation of measurement of symptom clusters” and “the evaluation of the potential to use large data sets and electronic health records to evaluate symptom clusters”. The fifth expert group also defined some directions for future research, one of which was to “apply new analytic techniques to symptom cluster research”. The international expert panel noted that the investigation of symptom clusters may result in the identification of causal relationships between symptoms, possibly informing targeted interventions on symptoms with a causal force [10].

Currently, we are in limbo of the explorative phase regarding symptom cluster research, where universal symptom clusters require identification. The inconsistencies in symptom clusters as identified by the aforementioned systematic reviews [9, 11] and corroborated by the international panel [10] are preventing the field of symptom cluster research to move into confirmatory research. The current study hopes to contribute to identifying symptom clusters by using a robust and innovative explorative analysis method.

Previous symptom cluster research is predominated by methods focused on latent variable (mixture) modeling. This method classifies individuals into unobserved clusters with (more) homogenous patterns within the clusters. Models based on homogeneity have the drawback that if most individuals are relatively alike, very large clusters will be identified. The previous literature [9, 11] indicates that most cancer patients are all very sick. This often creates a “general sickness” cluster. However, to identify symptom clusters we strive to identify the subclusters within such a larger cluster. Density-based clustering methods are designed to identify clusters of varying densities. As such, these methods are particularly useful in separating smaller subclusters (with higher densities) from larger clusters (with lower densities). A recent development in the field of density-based clustering methods is Hierarchical Density-Based Spatial Clustering of Applications with Noise (HDBSCAN) [12].

One further limitation to previous symptom cluster research is the limited breadth of symptoms studied [9]. Research has shown that cancer patients and survivors suffer a broad range of symptoms and other health issues [13–17]. These other health issues include lifestyle behaviors such as smoking or psychosocial constructs such as heightened stress, that do not fit into a semantic definition of ‘symptoms’. Nevertheless, they are likely interrelated and contribute to the disease profile of patients and survivors. As such, the current study sought to include these constructs, by clustering both symptoms and health determinants.

The aim of this study is to investigate symptom clusters among cancer patients and survivors, by analyzing a large dataset of a broad range of self-reported symptoms using HDBSCAN [12]. The results of the present study will contribute to the establishment of a conceptual framework and an approach for the evaluation of symptom and health determinants cluster measurements.

## Methods

### Study population

The study sample consisted of users of the eHealth application Oncokompas [13]. These users are Dutch cancer patients (currently undergoing treatment) or cancer survivors (treatment has ended) who are/were treated with curative intent. The application is available for patients and survivors through referral of their main healthcare provider. In total, data of 1032 users were included, who consented to the use of their data for research purposes. Of these users, 715 users of Oncokompas were referred by a healthcare provider in routine care, 191 cancer survivors were invited to participate in a randomized controlled trial (RCT) investigating the efficacy of Oncokompas [13], 72 colon cancer survivors were invited to participate in a multi-centre RCT [14], and 54 breast cancer survivors were invited to participate in a pilot on the feasibility of Oncokompas [15].

### Materials

Oncokompas is a fully automated online application that supports cancer survivors in their self-management by 1) monitoring their health-related quality of life (HRQOL) and (cancer-generic and tumour-specific) symptoms and health determinants; and 2) providing tailored feedback on their scores with a personalized overview of supportive care options, with the aim to reduce symptom burden and improve HRQOL [13]. Oncokompas covers a total of 46 topics on five generic domains applicable for all cancer survivors: physical, psychological, and social HRQOL, healthy lifestyle, and existential topics; and 29 tumour-specific topics for survivors of breast cancer, colorectal cancer, head and neck cancer, and lymphoma. Users can choose which topics they wish to fill in. In the current study, only the generic topics were used. Oncokompas consists of three components: ‘Measure’, ‘Learn’, and ‘Act’. For the current study, only the Measure component is of interest, the Learn and Act component are detailed elsewhere [13]. In the Measure component, users can independently complete patient reported outcome measures (PROMs) targeting the selected topic(s). On each of the selected topics, the user receives a green (no well-being risk), orange (moderate well-being risk), or red (high well-being risk) outcome. The current study focuses on 26 of the 46 generic topics, as these 26 topics represent physical or psycho-social symptoms and health determinants that often occur based on literature [16]. Table 1 details the symptoms and health determinants, and PROMs that were used in the analysis, as well as the possible color outcomes on each PROM. Each symptom and health determinant consists of one or multiple PROMs, which were selected by the project team in collaboration with expert teams and based on Dutch practical guidelines (from the Netherlands Comprehensive Cancer Organisation) and literature searches [17].

**Table 1:** Overview of Oncokompas topics

Topics	PROM	Possible scores
Contact with doctor	EORTC IN-PATSAT32	Green; Orange
Dedication to work	Visual Analogue Scale	Green; Orange
Smoking	Oncokompas expert-based questionnaire	Green; Orange; Red
Alcohol use	Alcohol 5-shot	Green; Orange; Red
Relaxation	Perceived Stress Scale	Green; Orange; Red
Physical activity	Oncokompas expert-based questionnaire	Green; Orange
Body weight	BMI & Short Nutritional Assessment Questionnaire	Green; Orange; Red
Physical limitations daily life	Patient Specifieke Klachtenlijst (Dutch-specific)	Green; Orange; Red
Insomnia	Insomnia Severity Index	Green; Orange; Red
Fatigue	Numeric Rating Scale	Green; Orange; Red
Pain	Numeric Rating Scale	Green; Orange; Red
Constipation	Numeric Rating Scale	Green; Orange; Red
Diarrhea	Numeric Rating Scale	Green; Orange; Red
Lack of appetite	Numeric Rating Scale	Green; Orange; Red
Nausea or vomiting	Numeric Rating Scale	Green; Orange; Red
Shortness of breath	Numeric Rating Scale	Green; Orange; Red
Hearing problems	Caron hearing questionnaire	Green; Orange; Red
Tinnitus	Oncokompas expert-based questionnaire	Green; Orange; Red
Psychological complaints	Hospital Anxiety and Depression Scale	Green; Orange; Red
Memory / concentration	SF-36 ‘cognitive functioning’	Green; Orange; Red
Social life	De Jong-Gierveld Loneliness Scale	Green; Orange; Red
Financial problems	EORTC QLQ-C30 ‘financial problems’	Green; Orange; Red
Intimacy and sexuality	Female Sexual Function Index (women) / International Index of Erectile Function (men)	Green; Orange; Red
Body image	Body Image Scale	Green; Orange; Red
Relationship with partner	Dyadic Adjustment Scale Short Form	Green; Orange; Red
Relationship with children	Vragenlijst Gezinskernmerken Short Form (Dutch-specific)	Green; Orange; Red

### Data analysis

Data of cancer survivors who used Oncokompas up to April 29th 2019 were used. Users can fill in Oncokompas more than once. Unfortunately, not enough users filled in Oncokompas more than once to be able to analyze trends over time. To remove within-user variance, when users had filled in Oncokompas more than once, one random time point was selected of the user for the current study. Sensitivity analyses were run by repeating the analysis a number of times using different random seeds for row selection. The results did not differ regarding the main results, and will not further be discussed. All analyses were run in R version 3.5.3 [18], or in Python version 3.7.1 [19]. Two types of analyses were used: network analysis and cluster analysis.

For the cluster analyses, HDBSCAN [12] was performed using the hdbscan library in Python [20]. HDBSCAN separates a dataset into clusters of high and low density. HDBSCAN is an extension of the DBSCAN clustering algorithm [21], where HDBSCAN is capable of identifying clusters of varying densities and is more robust to parameter selection [20]. This makes the HDBSCAN algorithm particularly useful in separating smaller subclusters (with higher densities) from larger clusters (with lower densities). Data points that do not fit into any of the identified clusters are labeled as noise by the algorithm. The Jaccard distance metric was used due to the categorical nature of our measurement of a symptoms and health determinants. The minimum points required to form a cluster (minimum cluster size) was set to 26 (number of modules). Because we were interested in subclusters of symptoms and health determinants, the minimum sample was set to 1, and leaf clustering was used for cluster selection. These parameters prioritize the extraction of multiple smaller rather than larger clusters. Due to the explorative nature of the analysis we did not perform hyperparameter tuning to find the parameters that lead to the least amount of noise. The parameters resulting from hyperparameter tuning may not be able to extract the subclusters with higher densities that we are interested in, instead extracting the larger clusters with lower densities.

The network analyses were performed using the tidygraph [22] and ggraph [23] packages in R. The network graphs were nondirectional, and edges were calculated as the raw number of connections between nodes (i.e. the occurrence of symptom-pairs among the same patient). Weighted degree centrality was calculated using the edges as weights.

The choice was made to perform the clustering based on the presence of a symptom or health determinant, instead of a continuous score of severity. Methodologically, clustering based on a dichotomous presence of a symptom or health determinant results in clusters that are more easily interpretable (i.e. “all the patients in this cluster have this symptom”) than clusters based on symptom severity (i.e. “these patients share a similar range of symptom severity”). The benefit of a more easily interpretable cluster is two-fold. Firstly, it will hopefully lead to more easily replicable symptom and health determinant clusters in follow-up confirmatory research. Secondly, the knowledge regarding identified symptom and health determinant clusters it will hopefully be more useful in practice. We theorize it is easier for clinicians to relate the presence of symptoms and health-related issues to one another, than it is to relate the severity of such symptoms and health-related issues to one another. For example, it appears easier to identify the probability of insomnia being present when a patient has been diagnosed with depression, rather than the probability of insomnia being present based on a severity scale of depression.

One analysis was run on only high risk (red) scores as the definition of a symptom or health determinants being present, as these scores are based on cut-off scores with most empirical evidence. Three symptoms on which a high risk scores was not possible (see Table 1) were excluded for this analysis. While severity was not chosen as the operationalization of a symptom or health determinant, it may be an influence on how symptoms and health determinants cluster together [9]. Consequently, a second analysis was run on moderate-to-high risk (orange and red) scores as the definition of a symptom or health determinant being present, which also included the previously excluded symptoms and health determinants.

## Results

### Patient characteristics

Table 2 shows the patient characteristics. The mean age was 61.5 years (range 25–88), the majority was female (68%), approximately half was treated for breast cancer (49%), most had completed treatment (60%), and most patients were treated with surgery (79%).

**Table 2:** Descriptive statistics

	Mean	SD		N	%
Age	60.52	11.31			
Gender			Female	701	67.99%
			Male	330	32.01%
Education			Elementary school	24	2.33%
			High school	164	15.91%
			Vocational education	590	57.23%
			College	115	11.15%
			University	107	10.38%
			Post-doctoral	23	2.23%
			Other	8	0.78%
Cancer type			Breast cancer	504	48.88%
			Colon cancer	182	17.65%
			Lymphoma	73	7.08%
			Head and neck cancer	60	5.82%
			Rectal cancer	40	3.88%
			Other	39	3.78%
			Lung cancer	31	3.01%
			Prostate cancer	29	2.81%
			Gynecologic cancer	20	1.94%
			Bladder or kidney cancer	17	1.65%
			Skin cancer	11	1.07%
			Blood cancer	9	0.87%
			Esophageal cancer	5	0.48%
			Brain cancer	4	0.39%
			Pancreatic or liver cancer	4	0.39%
			Stomach cancer	3	0.29%
Treatment status			Treatment completed	614	59.55%
			Currently being treated	172	16.68%
			Not yet treated	94	9.12%
			Unknown	80	7.76%
			No treatment	71	6.89%
Treatment type			Surgical	743	79.04%
			Chemotherapy	103	10.96%
			Radiation	38	4.04%
			Chemoradiation	24	2.55%
			Other	10	1.06%
			Hormone therapy	7	0.74%
			Wait-and-see	7	0.74%
			Immunotherapy	4	0.43%
			Unknown	4	0.43%

### High risk score analysis

In the high risk score analysis, seven clusters were extracted. A total of 393 data points were deemed noise, which amounted to 19.31% of the data, which is a non-negligible amount.

The cluster profiles are presented in Table 3. The cell numbers represent how many patients with a certain symptom or health determinant were present in any given cluster. The largest cluster (cluster 7), represents a “general sickness cluster”, encompassing patients who suffer from most symptoms and health determinants that were present in the data set. Next are two clusters that represent patients who experienced one symptom almost exclusively: a psychological complaint cluster (cluster 1), and a physical limitations cluster (cluster 2). Cluster 3 represents patients who mainly experienced symptoms and health determinants regarding body weight, alcohol use, and social life, while cluster 4 represents patients who mainly experience symptoms and health determinants regarding physical limitations, intimacy/sexuality, and body weight. Cluster 5 is the second-largest cluster with regard to number of symptoms and health determinants, and represents patients who experienced psychological symptoms and health determinants, and various physical symptoms. Lastly, cluster 6 represents patients who experienced psychological complaints, problems with relaxation, and social life.

**Table 3:** High risk cluster profiles

Symptom/Health Determinant	Noise	Cluster 1	Cluster 2	Cluster 3	Cluster 4	Cluster 5	Cluster 6	Cluster 7
Psychological complaints	225	65		1	6	36	30	52
Fatigue	114			1	6	21	1	48
Physical limitations daily life	100		27		18	33	5	46
Memory / concentration	53					1	3	26
Relaxation	123					1	31	25
Social life	82			12		9	29	24
Insomnia	66			1	1	3	2	21
Pain	64			1		10	1	19
Intimacy and sexuality	74			1	15	11		15
Hearing problems	107				4	26	1	15
Shortness of breath	47				2	15	1	9
Body weight	35			22	14	3		8
Constipation	25						3	3
Smoking	41			3		6	1	2
Diarrhea	33							2
Body image	15	3						1
Relationship with partner	21					1	4	1
Alcohol use	22			12	2		2	
Lack of appetite	23							
Relationship with children	19							
Nausea or vomiting	18							
Financial matters	17							
Tinnitus	6							

*Note*: The cell numbers represent the number of patients with a certain symptom / health determinant in any given cluster. Noise indicates data points that do not fit into any of the identified clusters

**Fig. 1:**
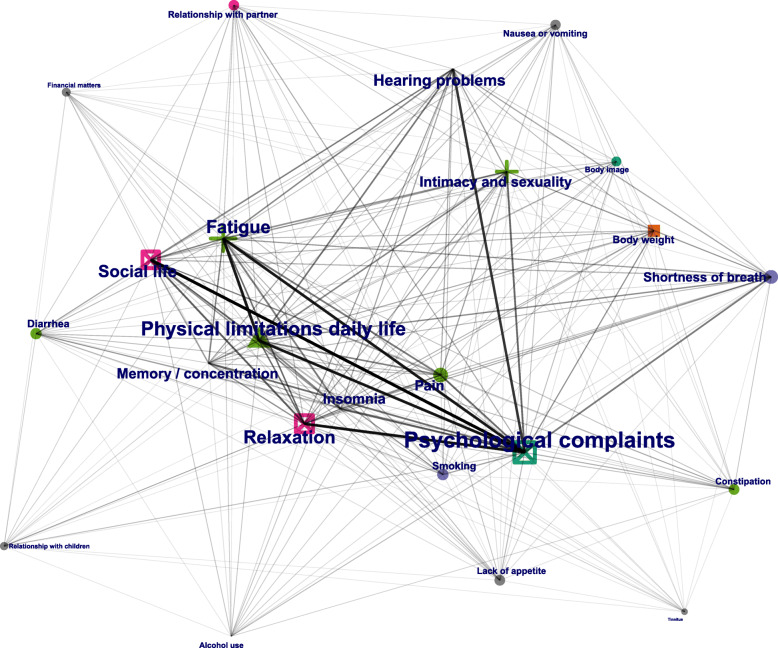
Network plot high risk score analysis. Every point in the network plot represents a symptom or health determinant as defined in Table 1. The size of the points represents the amount of connections it has with other symptoms and health determinants (weighted degree centrality). The thickness of the lines represents the amount of connections between those two specific symptoms or health determinants. Symptoms and health determinants are colored based on their main cluster (the cluster in which the symptom or health determinant is most frequent), and shapes are based on their subcluster (the cluster in which the symptom or health determinant is second-most frequent, with a minimum frequency of 5)

Figure 1 shows the network plot of the main analysis. The plot shows both the main cluster of each symptom or health determinant (the cluster in which the symptom or health determinant is most frequent), as well as the subcluster of each symptom or health determinant (the cluster in which the symptom or health determinant is second-most frequent, with a minimum frequency of 5). Psychological complaints and physical limitations have the highest weighted degree centrality and are connected to nearly all symptoms and health determinants. The intra-cluster connections range from large (mostly connections originating from psychological complaints or physical limitations), to moderate (most intra-cluster connections), to small (mostly connections originating from fringe symptoms or health determinants with less neighbors).

### Moderate-to-high risk score analysis

In the moderate-to-high risk score analysis, three clusters were extracted. A total of 579 data points were deemed as noise, which amounted to 39.25% of the data, which is a high amount.

The cluster profiles are presented in Table 4. One small cluster was extracted with patients who only experienced health determinants regarding body weight (Cluster 1). Two large clusters emerged: a lifestyle and psychosocial cluster (cluster 2), and a physical symptoms cluster (Cluster 3).

**Table 4:** Moderate-to-high risk cluster profiles

Symptom/Health Determinants	Noise	Cluster 1	Cluster 2	Cluster 3
Physical activity	257		175	130
Relaxation	105		88	67
Insomnia	99		5	67
Contact with doctor	75		46	66
Shortness of breath	79		1	65
Physical limitations daily life	100		2	51
Fatigue	136		3	50
Social life	58		57	43
Alcohol use	147		98	39
Tinnitus	85		3	39
Pain	59			38
Financial matters	71		52	36
Lack of appetite	26			25
Intimacy and sexuality	58		2	24
Relationship with partner	39		19	18
Psychological complaints	96		74	16
Constipation	26		1	16
Nausea or vomiting	16			16
Hearing problems	26		3	11
Memory / concentration	13			11
Body weight	62	18	123	10
Relationship with children	36		32	10
Body image	16			10
Dedication to work	28		1	8
Diarrhea	5			4
Smoking	15		7	2

*Note*: The cell numbers represent the number of patients with a certain symptom or health determinant in any given cluster. Noise indicates data points that do not fit into any of the identified clusters)

**Fig. 2:**
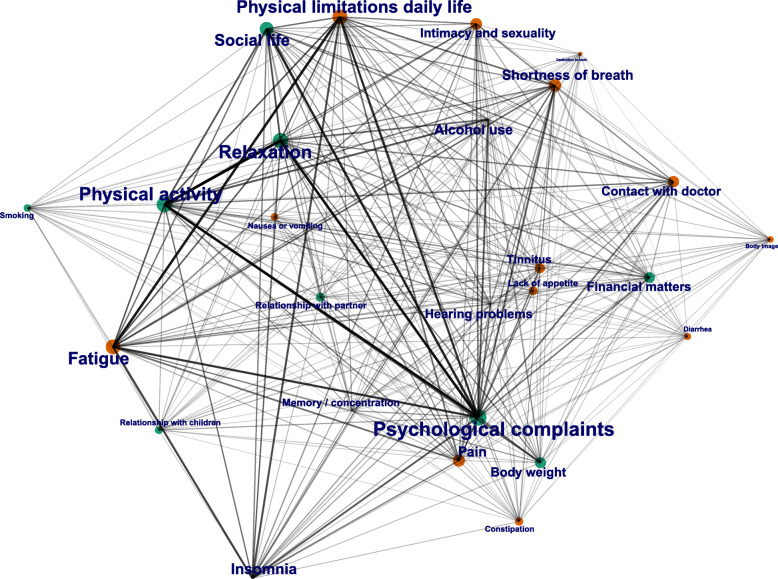
Network plot moderate-to-high risk score analysis. Every point in the network plot represents a symptom/health determinant as defined in Table 1. The size of the points represents the amount of connections it has with other symptoms and health determinants (weighted degree centrality). The thickness of the lines represents the amount of connections between those two specific symptoms or health determinants. Symptoms and health determinants are colored based on their main cluster (the cluster in which the symptom or health determinant is most frequent)

Figure 2 shows the network plot of the sensitivity analysis. The plot does not show the body weight cluster, as this health determinant was strongly incorporated into the psychosocial and lifestyle cluster. Psychological complaints, physical limitations, physical activity, relaxation, and fatigue have the highest weighted degree centrality and are connected to nearly all symptoms and health determinants. The intra-cluster connections range from large (mostly connections originating from the symptoms and health determinants with high weighted degree centrality), to moderate (most intra-cluster connections), to small (mostly connections originating from fringe symptoms and health determinants with less neighbors). This network analysis shows more inter-cluster connections than the main analysis. Strong connections exist between psychological complaints (cluster 2) and fatigue (cluster 3), and physical limitations (cluster 3). Moderate connections exist between psychological complaints (cluster 2) and intimacy/sexuality (cluster 3), pain (cluster 3), and insomnia (cluster 3). Moderate connections exist between physical limitations (cluster 3) and social life (cluster 2), physical activity (cluster 2), relaxation (cluster 2), and social life (cluster 2).

## Discussion

In this explorative study we used HDBSCAN to extract symptom and health determinant clusters based on scores on PROMs of cancer patients/survivor who used the eHealth application Oncokompas. When analyzing the high risk scores of patients, different clusters appeared compared to the analysis of moderate-to-high risk scores. When analyzing patients showing high-risk scores, we found one overarching cluster containing most symptoms and health determinants measured, as well as six subclusters. When analyzing patients showing moderate-to-high risk scores, we found two overarching clusters: one representing psychosocial symptoms and health determinants, and one representing physical symptoms and their health consequences.

This study was explorative in nature, and while the symptoms and health determinants used in the current study do not entirely line up with all symptoms reported on by previous research [5, 7–9, 11], it is of interest to see whether symptom and health determinant clusters identified in our dataset line up with the symptom clusters previously identified. First, a fatigue-depression-pain cluster [9, 11] has been reported by previous studies. We found fatigue, psychological complaints, and pain to be clustered together in the “general sickness” cluster as well as in the “physical symptoms and consequences” cluster in the high risk score analysis. And while fatigue and pain clustered in the “physical symptoms and consequences” cluster in the moderate-to-high risk score analysis, they were not clustered with psychological complaints.

Second, previous literature showed evidence for a depression-anxiety-insomnia cluster [5, 9]. In the present study, insomnia and psychological complaints were clustered together in the “general sickness” cluster, and showed a moderate connection in the network analysis (weight = 76) in the high risk score analysis. In the moderate-to-high risk score analysis insomnia and psychological complaints were not clustered together, but did show a strong connection in the network analysis (weight = 219).

Third, a psychological symptom cluster was found in multiple previous studies [9, 11]. In the high risk score analysis we found both a “psychological complaints” and “psychosocial” cluster. However, in the moderate-to-high risk score analysis we found a broader psychosocial and lifestyle cluster.

Fourth, a pain-fatigue-insomnia-lack of appetite clusters was reported [11]. In the high risk score analysis, pain, fatigue, and sleeping issues clustered together in the “general sickness” cluster; and pain and fatigue clustered in the “physical symptoms and consequences” cluster. Meanwhile, in the moderate-to-high risk score analysis, pain, fatigue, sleeping issues, and lack of appetite clustered in the “physical symptoms and consequences” cluster.

Fifth, a specific association between cognitive functioning and psychological distress was found in previous literature [7]. In our high risk score analysis, memory/concentration and psychological complaints were clustered together in the “general sickness” cluster, and showed a moderate connection in the network analysis (weight = 66); while in the moderate-to-high risk score analysis memory/concentration and psychological complaints were not clustered together, but did show a moderate connection in the network analysis (weight = 98).

Sixth, literature showed a specific association between memory/concentration and fatigue [7]. In our high risk score analysis, memory/concentration and fatigue clustered in the “general sickness” cluster, and showed a small to moderate connection in the network analysis (weight = 54). In the moderate-to-high risk score analysis, memory/concentration and fatigue clustered in the “physical symptoms and consequences” cluster, and showed a moderate connection in the network analysis (weight = 93).

Seventh, a specific association between sexual problems and body image has been reported [8]. Intimacy/sexuality and body image were not clustered together in our high risk score analysis, as body image was not part of any cluster, and showed a very weak connection in the network analysis (weight = 5). In the moderate-to-high risk score analysis, intimacy/sexuality and body image clustered in the “physical symptoms and consequences” cluster, but showed a weak connection in the network analysis (weight = 22). These results may be explained by the fact that we have very few patients in the main data set with body image problems.

Eight and last, a specific association between sexual problems and psychological distress was found previously [8]. In our high risk score analysis, intimacy/sexuality and psychological complaints were clustered together in the “general sickness” cluster as well as in the “physical symptoms and consequences” cluster, and showed a moderate connection in the network analysis (weight = 73). In the moderate-to-high risk score analysis, intimacy/sexuality and psychological complaints were not clustered together, but did show a strong connection in the network analysis (weight = 159).

These results show that many of the previously reported (sub) clusters were found in our high risk score analysis, but not in the moderate-to-high risk score analysis. There may be an inherent difference on the co-occurence of symptoms and health determinants dependent on severity. For patients with higher severity, we observed more connections between the physical and psycho-social symptoms and health determinants, compared to patients with lower severity. This provokes the question of causality: do patients with higher severity of physical issues develop higher severity of psycho-social issues, vice versa, or are both higher severities developed in tandem due to a third causal force? The statistical methods we used are associative, intending to identify clusters of co-occuring symptoms and health determinants which do not necessarily share the same etiology [10]. As such, we cannot offer an answer to the question of causality. But future research could use methodology more suited for such investigations.

It has been suggested that cluster symptoms are not the same across different cancer diagnoses [11]. The current study did not perform subgroup analyses between cancer diagnoses, as the corresponding sample sizes would not have been sufficient for all diagnoses. In future research, after Oncokompas has attracted more users of differing diagnoses, such subgroup cluster analyses may provide further insights into this possibility.

While many studies researching symptom clusters pre-dominantly use variations of exploratory and confirmatory factor analyses [9, 11], innovation in methodology is starting to gain traction. The current study made use of an explorative machine learning algorithm and a rather simple method of network analysis. Other studies innovated through the use of new and more complex network analysis methods: Pairwise Markov Random Field (PMRF) [24] and concordance networks [25]. These methods create more robust networks of symptoms, but the estimation of clusters is inherently different than through the use of a specific cluster algorithm. The clusters (i.e. *communities* in network terminology) are defined through the WalkTrap [24] or random walk [25] algorithms which identifies clusters of network nodes (i.e. symptoms) that are highly connected (i.e. higher valued edges). To put it more simply: while the current study identifies symptom and health determinant clusters based on the density of co-occuring symptoms, the network methodology identifies symptom clusters based on the strength of network edges.

As these are two distinctly different ways of defining what a cluster entails, it is of interest to see whether cluster machine learning algorithms and advanced network analyses reveal similar clusters. A comparison between the results of the current study and the aforementioned studies shows some overlap in the identified clusters. The study using PMRF found six main clusters: psychological symptoms, hormonal symptoms, respiratory symptoms, nutritional symptoms, CTX-related symptoms, and pain and abdominal symptoms [24]. Particularly noteworthy is the separation between psychological and physical symptoms. The study using concordance networks found three major clusters: Fatigue/Constipation, Fatigue/Sleep, and Fatigue/Sleep/Neck Spine [25]; where the only overlap with the current results can be found in the Fatigue/Sleep cluster.

Whether this is due to the analytical method, the measurement method of symptoms, differences in population, or other factors is impossible to determine at this time. It would be of great interest for future research to investigate how resulting clusters differ between various machine learning clustering algorithms (e.g. HDBSCAN, DBSCAN, GMM), various network analyses (e.g. PMRF, concordance networks), and various more traditional clustering analyses (e.g. EFA, CFA). Research focused on comparing analytical methodology can use data simulation methods to validate which methods reproduce the most accurate clusters as the investigators directly control the existence of clusters. A data simulation approach can be supplemented with a comparison of results from real data to provide a holistic view of the results of differing clustering methods. Such a comparison can inform the previously identified objective of identifying robust “new analytic strategies for symptom cluster research” by the 2015 expert panel [10].

One strength, but simultaneously a limitation, of this study is the use of different measurement tools for each separate symptom and health determinant. It has been argued that a standardization of how to measure symptoms for use in classifying symptom clusters is necessary for reproducible and valid interpretations [10]. However, the use of multiple (standardized and validated) measurement instruments creates the possibility to analyse many more symptoms and health determinants than would be possible when using only one standardized measurement tool.

There are three further limitations in regards to the way data was analyzed. First, while HDBSCAN was particularly suited for the current research question; due to the explorative nature of the analysis there is a distinct lack of easily interpretable fit metrics [12, 20]. While the proportion of noise provides us with information on the amount of data points that could not be allocated to a cluster, it is unclear when the amount of noise is too high. Second, by focusing on the extraction of smaller clusters we found a seemingly high noise count in the cluster analyses. This indicates that there may be other (likely larger) clusters that could be extracted with other parameter settings. The amount of noise data points could also likely be reduced by using an algorithm that searches for the optimal minimum cluster size (i.e. hyperparameter tuning). While such settings were judged not optimal to answer our particular research question, such analyses could improve the fit of the model to the data. Third, for users that filled in Oncokompas more than once, we chose a random data row to ensure that we did not increase bias in our data set. Another dataset (e.g. a non-random selection of the data row with most moderate-to-high risk scores) could have produced a different result.

The research line of symptom clusters may eventually lead to practical applications in patient care. Currently we find ourselves in the explorative phase where plausible clusters need to be identified through the use of robust explorative analysis techniques. To move towards a confirmatory phase of research, the identified symptom and health determinant clusters need to be replicated in samples using other measurement techniques as well as using confirmatory analysis techniques. Stable and replicable symptom clusters need to be identified. As such, it is of interest to see whether the symptom and health determinant cluster found in the current study can be replicated in other research.. A form of standardization on both measurement techniques and (confirmatory) analysis techniques has been argued [10], but which measurement and analysis techniques are most appropriate and should be the standard has not yet been firmly concluded. Through replication, information on comparability can be gained.

To move beyond the confirmatory phase, and towards a phase of practical implementation, causal modelling techniques could be used to investigate possible etiological connections between symptoms, as well as by using subgroup analyses for differing tumor types. In particular, an investigation of how these clusters may change over time is of interest for such causal analyses. Furthermore, implementation of risk and protective factors in future models can help identify which symptoms may be best targeted as well as how care for individual patients should be tailored [26].

Knowledge regarding symptom clusters may inform targeted interventions [10]. While the current study cannot attest to etiology or causality within the found symptom and health determinants cluster, the main finding of interest for clinicians is the association between physical symptoms and psycho-social symptoms and health determinants for patients facing severe symptoms and health determinant issues. As such, it is advisable to assess whether a patient may profit from psycho-social support when suffering from (multiple) severe physical symptoms, in addition to treatment of the physical symptoms themselves.

## Data Availability

The datasets generated and/or analyzed during the current study are not publicly available due to the sensitivity of health data and due to the fact that during the informed consent procedure participants did not consent to the publishing of generated data. Data are available from the corresponding author on reasonable request.
